# Integrated single-cell and spatial transcriptomic analyses reveal an aging-associated fibroblast subtype linked to tumor progression in human skin

**DOI:** 10.3389/fmed.2026.1800947

**Published:** 2026-07-08

**Authors:** Danping Pan, Haibin Wu

**Affiliations:** Outpatient Department, The Fifth People’s Hospital of Hainan Province, Haikou, Hainan, China

**Keywords:** aging, fibroblast, ScRNA-seq, sFRP2 and Wnt/β-catenin, spatial trancriptomics

## Abstract

Aging is a major risk factor for the development of many cancers, yet the tissue-level mechanisms linking physiological aging to tumor progression remain unclear. Although age-related accumulation of genetic mutations contributes to tumorigenesis, increasing evidence indicates that the aging tissue microenvironment also plays a critical role in shaping cancer susceptibility. In this study, we integrated single-cell RNA sequencing and spatial transcriptomic datasets from healthy human skin and major skin cancers, including melanoma, squamous cell carcinoma, and basal cell carcinoma, to define cell-type-specific aging programs and assess their representation in tumor-associated microenvironments. In healthy skin, aging was associated with widespread but cell-type-specific transcriptional remodeling across epithelial, immune, vascular, and stromal compartments, involving pathways related to inflammation, extracellular matrix organization, and intercellular communication. Projection of these aging signatures into skin cancer datasets revealed that aging-associated programs were not restricted to immune cells but were also prominent in stromal compartments. Age-adjusted survival analysis in melanoma further identified the fibroblast aging program as the most prominent stromal signature, with higher program activity being associated with poor patient outcomes, motivating a focused analysis of fibroblast remodeling in skin cancers. Within this fibroblast-centered framework, we found that SFRP2-expressing fibroblasts accumulated in aged skin and were also enriched in tumor-associated stroma, suggesting that this pre-existing fibroblast state may become incorporated into and potentially support the tumor microenvironment during skin cancer development. Spatial analyses in basal cell carcinoma and squamous cell carcinoma showed that SFRP2-associated fibroblast signals were enriched near tumor-associated regions and WNT-active niches. Additional spatial proxy analyses and expression profiling of matrix-remodeling and growth factor-related genes further supported the association of this fibroblast state with tumor-associated stromal remodeling. Together, our findings suggest that aging-associated fibroblast remodeling may contribute to a tumor-permissive skin microenvironment and provide a framework for understanding how aging tissue ecosystems influence skin cancer progression.

## Introduction

Skin is the largest organ in the body, serving as a critical barrier that protects against dehydration, ultraviolet (UV) radiation, and invasion by pathogen invasion (1, 2). It is a complex organ composed of diverse cell types, including keratinocytes, fibroblasts, melanocytes, and immunocytes, all of which work in concert to maintain tissue homeostasis. However, aging profoundly alters the structural, cellular, and molecular composition of the skin, reducing its regenerative capacity and resilience. Aging is also a well-established risk factor for a variety of diseases, including cancer (3, 4).

Skin cancer is among the most prevalent malignancies in the aging population, with basal cell carcinoma (BCC), squamous cell carcinoma (SCC), and melanoma being the most common types (5). Although the accumulation of genetic mutations has historically been considered the primary driver of cancer development, increasing evidence highlights the pivotal role of the tumor microenvironment (TME) in cancer initiation and progression (4). The TME comprises stromal cells and immunocytes that interact dynamically with cancer cells, influencing tumor growth, invasion, and response to therapy. In aging individuals, the TME undergoes significant alterations, including chronic inflammation, extracellular matrix (ECM) remodeling, and immunosenescence, all of which contribute to increased susceptibility to skin cancer (6).

Immunosenescence, the gradual deterioration of the immunological system with age, is a critical factor in the failure of the immunological system to recognize and eliminate tumor cells effectively. This age-associated decline includes reduced antigen presentation, impaired T-cell activation, and the accumulation of immunosuppressive cells, such as regulatory T cells and myeloid-derived suppressor cells (4, 7, 8). These changes create an immunological microenvironment that is less effective at tumor surveillance and more permissive to cancer progression. Additionally, stromal cells, particularly fibroblasts, undergo phenotypic and functional changes during aging that reshape the ECM and influence the behavior of surrounding cells (6, 9). Emerging technologies, such as single-cell RNA sequencing (scRNA-seq) and spatial transcriptomics (ST), now allow for unprecedented resolution in profiling cellular heterogeneity and gene expression changes in complex tissues (10, 11). These tools have illuminated age-related shifts in cellular composition and molecular pathways across a variety of tissues, including the skin (12). Despite these advancements, our understanding of how aging impacts specific cell types within the skin and how these changes contribute to cancer progression remains unclear.

In this study, we integrate single-cell RNA sequencing and spatial transcriptomics datasets to construct a comprehensive atlas of age-related changes in human skin and skin cancers. Our analyses reveal widespread age-associated transcriptional reprogramming across diverse stromal, immune, and epithelial compartments, highlighting both shared and tumor-type-specific aging trajectories. By linking these molecular programs to patient outcomes, we found that the fibroblast aging program showed the strongest association with survival, with higher signature activity being independently associated with poorer outcomes after controlling for patient age, sex, and tumor stage. This observation motivated our subsequent focus on age-associated stromal remodeling and SFRP2-marked fibroblasts in skin cancer. Together, these findings provide a system-level view of how aging reshapes the skin microenvironment, influences cancer vulnerability, and suggest potential therapeutic opportunities that incorporate the biology of aging.

## Methods

### Data acquisition and preprocessing

Publicly available single-cell RNA sequencing (scRNA-seq) datasets were downloaded from the NCBI Sequence Read Archive (SRA) and Genome Sequence Archive (GSA) using SRA Toolkit (v2.11.2) or Aspera Connect (v4.1.1) for high-speed data transfer (13). The dataset comprised skin samples from 14 female donors aged 18–76 years, previously published under GEO accession GSE130973 (14) and GSA accession HRA000395 (15). These datasets were merged, integrated, and processed for downstream analyses. Human melanoma scRNA-seq data were obtained from GEO accession GSE115978 (16). For squamous cell carcinoma (SCC), both scRNA-seq and spatial transcriptomics datasets were downloaded from GEO under accessions GSE218170 (17) and GSE208253 (18), respectively. For basal cell carcinoma (BCC), both scRNA-seq and spatial transcriptomics datasets were retrieved from ArrayExpress under accessions E-MTAB-13085 and E-MTAB-13084, respectively (19). For scRNA-seq datasets, the FASTQ files were aligned to the GRCh38 human genome reference using the Cell Ranger pipeline (v6.1.2). The resulting gene expression matrices were aggregated into a single matrix for downstream analyses. Cells with fewer than 200 detected genes, mitochondrial gene content exceeding 10%, or total gene counts greater than 2.5 standard deviations above the mean were excluded. To address batch effects and ensure robust integration across datasets, we systematically benchmarked three state-of-the-art data integration methods: LIGER, Scanorama, and Harmony (20–22). These methods were evaluated using several established metrics for both batch correction and biological signal preservation, including iLISI, kBET, silhouette scores, and graph connectivity. Based on comprehensive benchmarking results, we found that LIGER provided the most favorable balance between batch effect removal and retention of biological signal. Therefore, LIGER was selected as the integration strategy for all downstream analyses.

### Visium data processing

Raw Visium spatial transcriptomics data (BCL and FASTQ files) were processed using the Space Ranger v1.3.1 pipeline (10x Genomics). For each sample, gene-barcode matrices were loaded into R and organized using the SummarizedExperiment and SingleCellExperiment frameworks to retain spatial metadata, including histological image alignment and tissue position coordinates. Spots were initially filtered based on quality control metrics using the perCellQCMetrics function from the scran v1.14.3 package (23). Specifically, spots were excluded if they contained fewer than 200 detected genes, fewer than 500 total UMI counts, or more than 15% mitochondrial gene content. After quality filtering, gene counts were normalized using scran’s computeSumFactors, followed by log-transformation using logNormCounts from scater v1.14.3 (24).

#### Cell-type annotation

Cell types were annotated using a combination of automated clustering and manual curation. Initial clustering was performed with Seurat’s FindClusters function using a resolution of 0.5. Cluster identities were assigned based on canonical marker gene expression, including KRT14 (keratinocytes), COL1A1 (fibroblasts), PECAM1 (endothelial cells), and CD3D (T cells). Differentially expressed genes (DEGs) were identified using the Wilcoxon rank-sum test, with *p*-values adjusted for multiple testing using the Benjamini–Hochberg method. Marker genes were further validated by cross-referencing with published datasets (14, 15).

### Aging gene signature construction and validation

Cell-type-specific aging signatures were constructed from the healthy-skin scRNA-seq datasets. After cell-type annotation, cells from each major skin cell type were analyzed separately to avoid confounding age-associated changes with cell-type composition. Within each cell type, differential expression analysis was performed between young and old samples using the FindMarkers() function in Seurat with the Wilcoxon rank-sum test and Benjamini–Hochberg correction for multiple testing. Genes with adjusted *p*-values of < 0.05 and consistent directionality between age groups were retained as cell-type-specific aging-associated genes.

For each cell type, aging signature scores were calculated using the average expression of OLD-upregulated genes relative to OLD-downregulated genes. These scores were used to evaluate whether the derived signatures tracked chronological age across healthy-skin samples. The complete cell-type-specific aging gene lists are provided in Supplementary Table S2.

### Trajectory analysis

Single-cell trajectory analysis was performed using Monocle 3 (25) to infer developmental trajectories of fibroblasts, enabling the identification of transitional states leading to SFRP2-positive fibroblasts. The pseudotime dynamics of aging-associated gene expression were visualized to assess how fibroblast subtypes evolve with age.

### Ligand–receptor interaction analysis

Cellular communication networks were analyzed using the CellChat package (26), focusing on ligand–receptor interactions mediated by fibroblasts. Pathway enrichment analysis identified key signaling axes. The signaling strength was quantified and visualized as circular plots for young, middle-aged, and old skin samples.

### SCENIC analysis

To infer transcription factor (TF) activity, we applied the pySCENIC pipeline (v0.11.2) (27) to epithelial cells from SCC scRNA-seq datasets. Gene regulatory networks were inferred using GRNBoost2, followed by motif enrichment analysis with cisTarget (hg38, v9). Regulon activity scores were computed via AUCell.

### Prognostic analysis

Prognostic implications of aging-associated gene programs were assessed using TCGA-SKCM bulk RNA-seq and clinical data. For each cell-type-specific aging signature, patients were stratified into high- and low-score groups using the median signature score. Kaplan–Meier curves were used for visualization. To test whether the association between aging signatures and survival was independent of chronological age, multivariable Cox proportional hazards models were fitted with either signature group or continuous signature score as the predictor, adjusting for patient age, sex, and tumor stage.

### Statistical analysis

Statistical analyses were conducted using R (v4.2.2) and Python (v3.9). DEGs were identified with a threshold of adjusted *p*-value of <0.05 and absolute log2 fold-change of >0.25. Batch correction and integration metrics were calculated using the scIB framework. All plots, including UMAPs, heatmaps, and spatial transcriptomics maps, were generated using ggplot2, ComplexHeatmap, and Seurat visualization tools.

### Code and data availability

All custom scripts for preprocessing, integration benchmarking, cell-type annotation, aging signature construction, trajectory inference, ligand–receptor analysis, and survival analyses are openly available at our GitHub repository: https://github.com/BioGenYiShang/Aging_Fibroblast. Raw and processed single-cell RNA-seq and spatial transcriptomics datasets are publicly available from the original repositories, including healthy skin (GEO accession GSE130973 and GSA accession HRA000395), melanoma (GEO accession GSE115978), squamous cell carcinoma (SCC; GEO accessions GSE218170 for scRNA-seq and GSE208253 for Visium), and basal cell carcinoma (BCC; ArrayExpress accessions E-MTAB-13085 for scRNA-seq and E-MTAB-13084 for Visium); aligned gene expression matrices, intermediate objects, and analysis outputs generated in this study are available upon reasonable request to the corresponding author, subject to data storage limitations.

## Results

To characterize cell-type-specific transcriptional changes during human skin aging, we integrated two publicly available healthy-skin scRNA-seq datasets spanning 14 female donors aged 18–76 years. After quality control, 216,778 high-quality cells were retained for downstream analysis. We benchmarked multiple integration strategies, including LIGER, Scanorama, Harmony, and unintegrated analysis, using metrics that assessed both biological conservation and batch correction. LIGER showed the highest aggregate performance and was therefore used for downstream integrated analysis (Supplementary Figure S1A).

**Figure 1:**
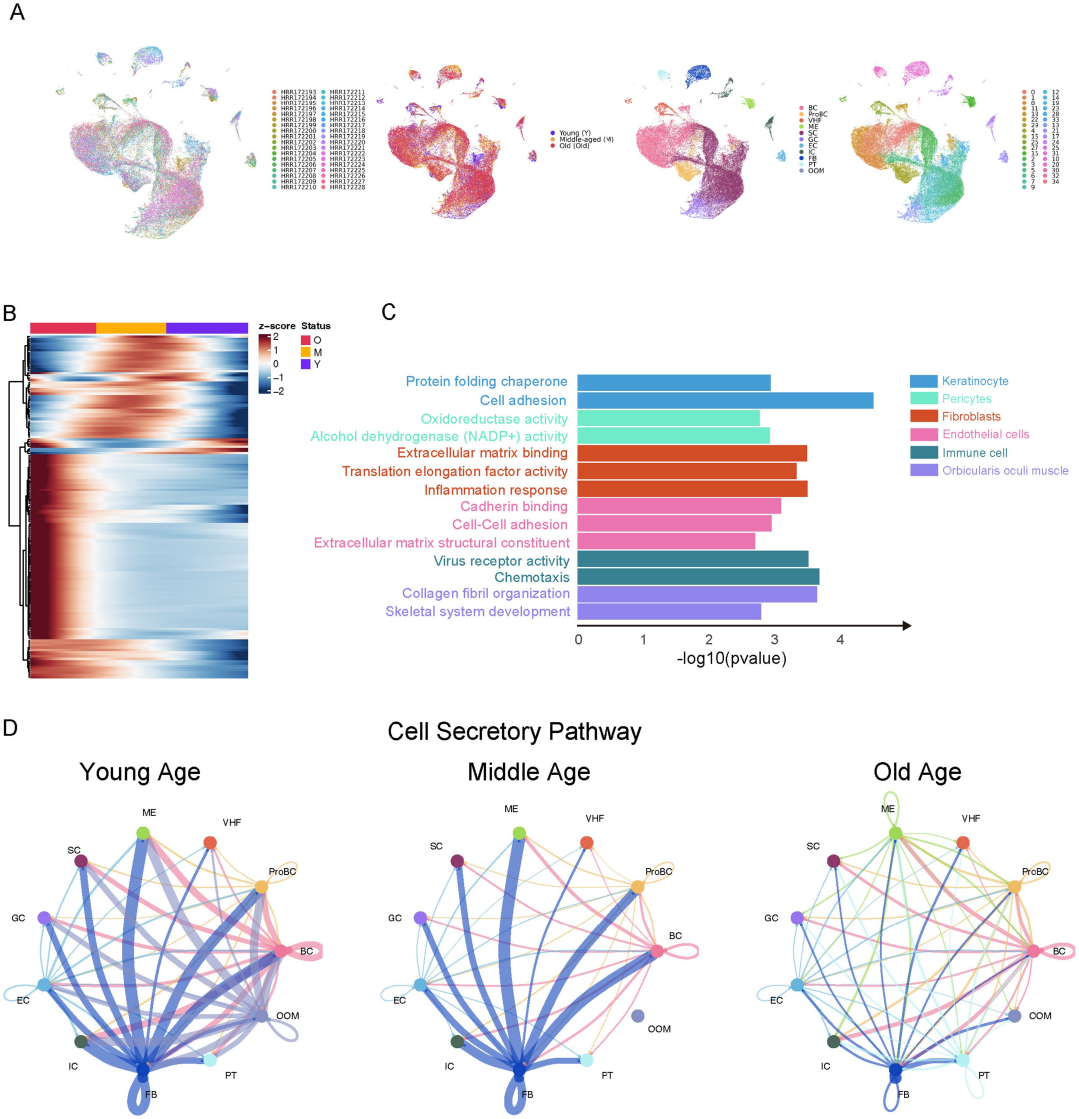
Single-cell RNA sequencing atlas of human skin aging. **(A)** UMAP visualizations of integrated healthy-skin single-cell RNA-seq data, showing major skin cell-type annotations, age group distribution, and quality control/read depth features. **(B)** Trajectory heatmap of age-associated gene expression across age-ordered cells. Columns are ordered by trajectory; age status is indicated above the heatmap, and color indicates z-scored gene expression. **(C)** Gene Ontology enrichment analysis of age-associated genes across major skin cell types. Bars show representative enriched biological processes, with colors indicating the corresponding cell type. **(D)** CellChat analysis of secreted signaling pathways in young, middle-aged, and old skin, showing age-associated remodeling of intercellular communication networks.

UMAP visualization of the integrated dataset resolved major skin cell populations, including keratinocyte subsets, melanocytes, endothelial cells, immune cells, fibroblasts, pericytes, and orbicularis oculi muscle cells (Figure 1A). Unsupervised clustering and donor composition analysis showed that clusters were not dominated by individual donors (Supplementary Figure S1B), while age group composition analysis revealed cell-type-specific changes across young, middle-aged, and old skin (Supplementary Figure S1C). Canonical marker gene heatmaps and dot plots further supported the assigned cell identities (Supplementary Figures S2A,C).

To define cell-type-specific aging-associated transcriptional programs, we compared young and old healthy-skin samples within each major skin cell type. This analysis identified aging-associated genes across multiple compartments, including epithelial, immune, vascular, and stromal cell populations. The number and magnitude of differentially expressed genes varied substantially across cell types, indicating that skin aging is not a uniform transcriptional process (Supplementary Figure S2B).

To visualize the behavior of these aging-associated genes, cells within each major cell type were ordered along an age-associated trajectory-like axis, and FindMarkers()-defined aging-associated genes were displayed as heatmaps (Figure 1B). The ordering was concordant with donor age groups, with young, middle-aged, and old cells distributed progressively along the axis. These results support that the identified signatures captured gradual age-related transcriptional remodeling rather than isolated donor-specific effects. Gene Ontology enrichment analysis of aging-associated genes highlighted biological processes related to immune activation, extracellular matrix organization, apoptotic signaling, and tissue remodeling (Figure 1C). We therefore examined whether aging was accompanied by altered cell–cell communication. CellChat analysis of secreted signaling pathways revealed age-associated remodeling of intercellular communication networks across young, middle-aged, and old skin (Figure 1D). Focused analysis of TNF, CXCL, and MIF signaling further showed age-associated changes in fibroblast-immune and macrophage-T-cell communication (Supplementary Figures S3A,B). Additional heatmaps of representative aging-associated genes in T cells and macrophages/DCs clarified the immune cell programs contributing to these age-associated changes (Supplementary Figures S3C,D).

### Cell-type-specific aging programs are activated in distinct skin cancers and are associated with patient prognosis

**Figure 2:**
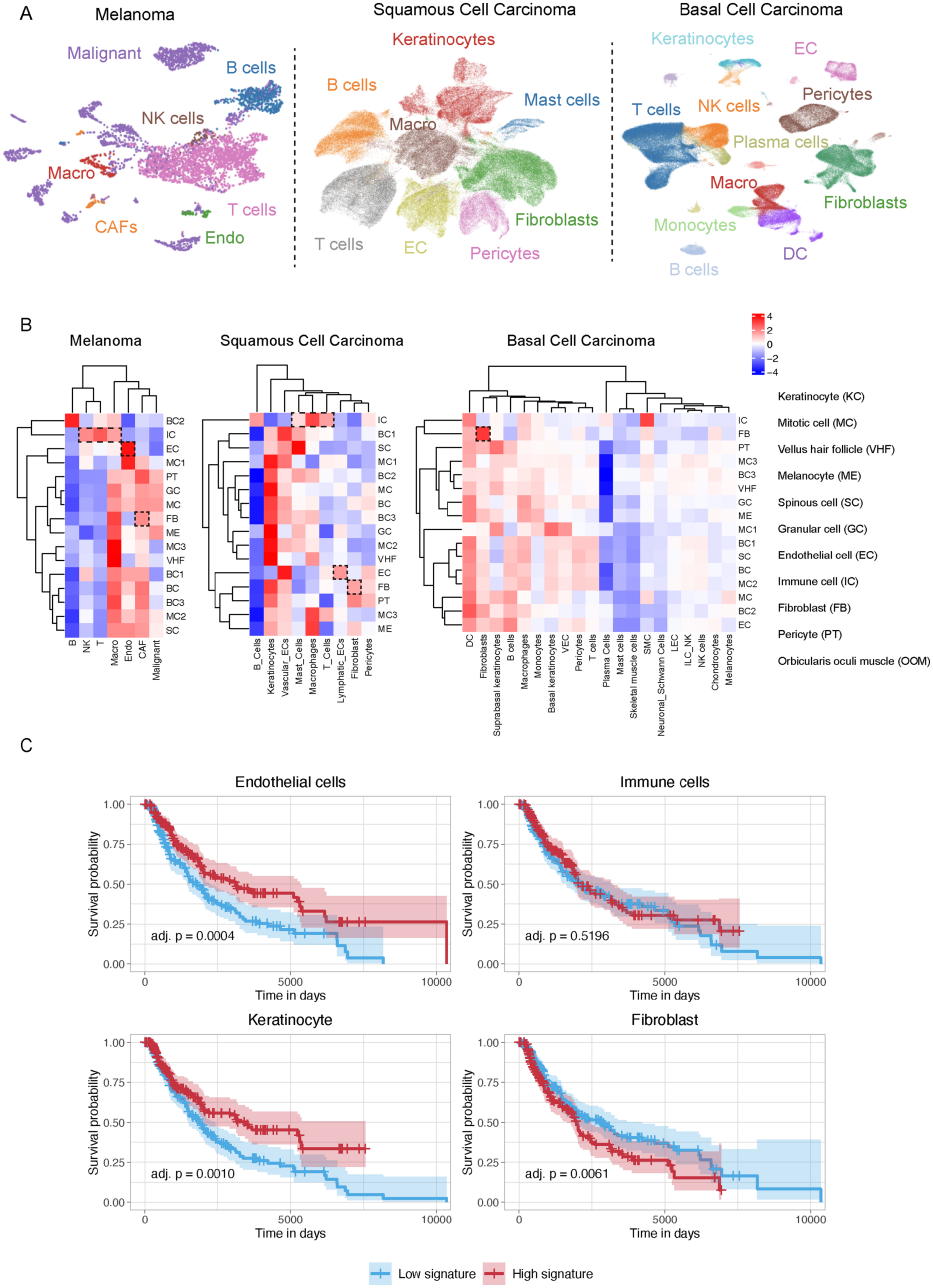
Cell-type-specific aging programs in skin cancers and their association with patient survival. **(A)** UMAP visualizations of public melanoma, squamous cell carcinoma (SCC), and basal cell carcinoma (BCC) single-cell RNA-seq datasets, colored by major tumor microenvironment cell types. **(B)** Heatmaps showing aging-signature scores across cell types in melanoma, SCC, and BCC. Cell-type abbreviations are shown with the corresponding full cell-type names. **(C)** Kaplan–Meier survival curves for TCGA-SKCM patients stratified by high or low aging signature activity in endothelial cells, immune cells, keratinocytes, and fibroblasts. Adjusted *p*-values are derived from multivariable Cox models controlling for patient age, sex, and tumor stage.

To investigate whether aging-associated programs defined in healthy skin are represented in skin cancer microenvironments, we analyzed public scRNA-seq datasets from melanoma, squamous cell carcinoma (SCC), and basal cell carcinoma (BCC). UMAP visualizations resolved major tumor microenvironment cell populations in each cancer type, including tumor/epithelial cells, immune cells, endothelial cells, fibroblasts, and other stromal populations (Figure 2A). We then projected the healthy-skin cell-type-specific aging signatures onto these cancer datasets to evaluate aging program activity across tumor-associated cell populations.

This analysis revealed cancer-type- and cell-type-specific patterns of aging program activity (Figure 2B). In melanoma, aging-associated programs were prominent in multiple compartments, including fibroblasts, endothelial cells, and immune cells. In SCC and BCC, aging program activity was more variable across cell types, with stromal compartments showing notable aging-associated signals. These findings indicate that aging-associated transcriptional programs are not restricted to immune cells in tumors but are also represented in fibroblast and vascular compartments.

Since chronological age itself could confound associations between aging signatures and clinical outcome, we first evaluated whether the curated aging signatures tracked donor age in healthy skin. Donor-level aging scores increased with chronological age across multiple cell types, supporting the use of these signatures as aging program readouts (Supplementary Figure S4A). We then assessed the prognostic relevance of cell-type-specific aging signatures in TCGA-SKCM. Kaplan–Meier curves were used for visualization, and multivariable Cox proportional hazards models were used to adjust for patient age, sex, and tumor stage (Figure 2C).

After adjustment for clinical covariates, the fibroblast aging signature showed the clearest association, with higher signature activity corresponding to poorer overall survival (Figure 2C). By contrast, other cell-type signatures showed weaker associations, non-significant effects after adjustment, or survival trends that were not consistently aligned with a high aging signature/poor outcome relationship. This age-adjusted analysis refined our interpretation of the cancer aging signatures and motivated a focused analysis of aging-associated fibroblast remodeling in skin cancer.

### SFRP2-expressing fibroblasts accumulate with age and are enriched in BCC tumor-associated stroma

Since the fibroblast aging signature showed the clearest age-adjusted association with poor outcomes, we next examined fibroblast states that could link aging-associated stromal remodeling to tumor-associated niches. SFRP2 was not used to define the aging signatures. Instead, we examined SFRP2-expressing fibroblasts as a fibroblast state emerging downstream of the fibroblast aging analysis. In healthy-skin fibroblasts, SFRP2 expression was increased in old compared with young fibroblasts, and a larger fraction of old fibroblasts expressed SFRP2 (Supplementary Figure S5A). These data suggest that SFRP2-expressing fibroblasts accumulate during skin aging and may become incorporated into tumor-associated stroma when cancer develops.

**Figure 3:**
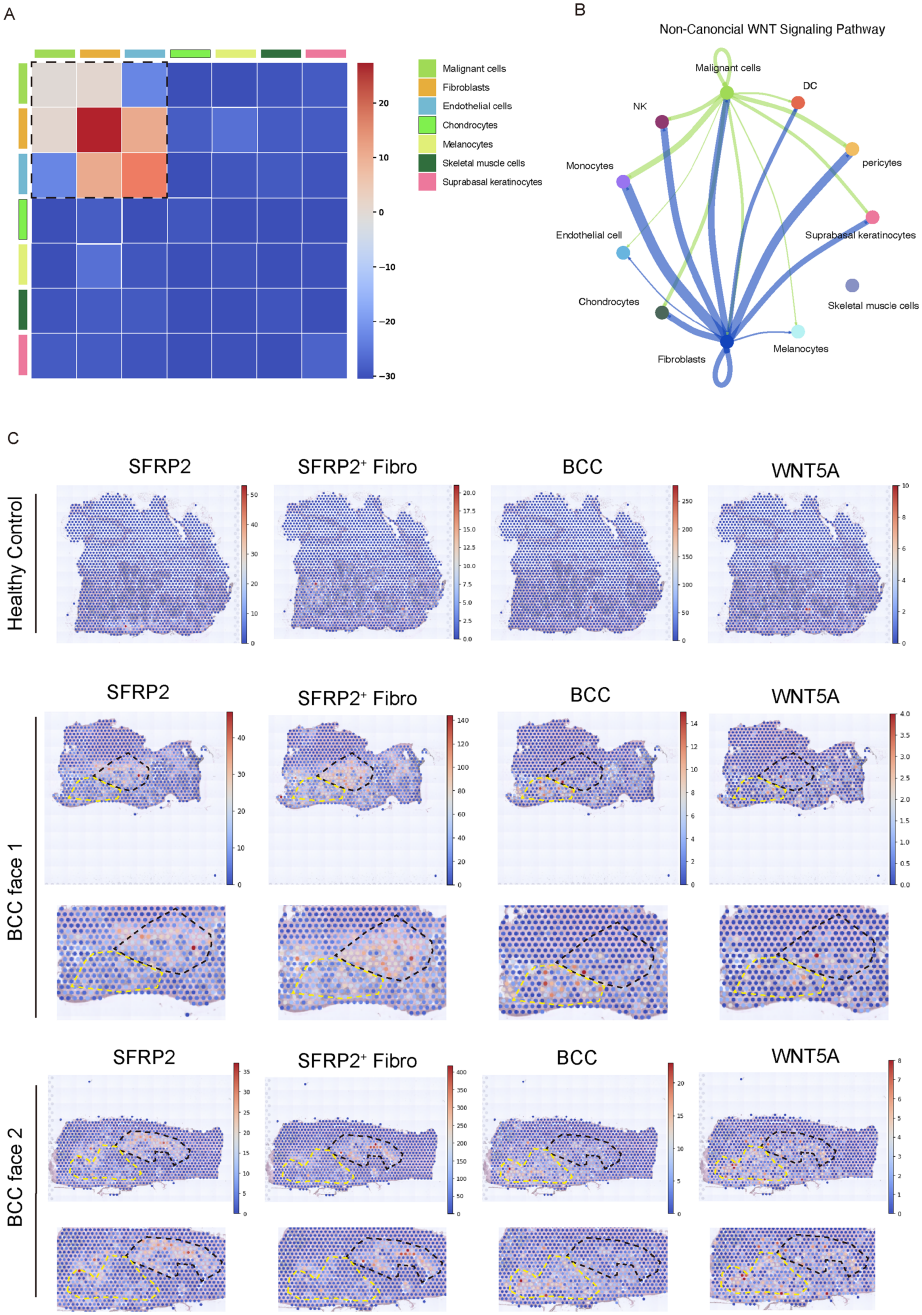
SFRP2-positive fibroblast programs are spatially associated with WNT-active niches in basal cell carcinoma. **(A)** Spatial proximity heatmap showing neighborhood relationships among BCC-associated cell types and tissue compartments. Values represent normalized spatial proximity scores. **(B)** Ligand–receptor network for the non-canonical WNT signaling pathway in BCC, highlighting fibroblast-associated communication and the SFRP2-LRP5/6 axis. **(C)** Spatial maps from healthy control and BCC tissue sections showing SFRP2 expression, inferred SFRP2-positive fibroblast abundance, BCC-associated signal, and WNT5A expression. Dashed outlines indicate tumor-enriched regions where applicable.

We then analyzed the spatial organization of SFRP2-associated fibroblast signals in basal cell carcinoma (BCC). Spatial proximity analysis of BCC Visium data revealed structured organization of tumor-associated and stromal compartments (Figure 3A). These values represent normalized spatial proximity scores rather than correlation coefficients. Fibroblast-associated regions showed strong proximity to endothelial-associated stromal compartments, whereas cancer cell-fibroblast proximity was close to the normalized baseline. Thus, the BCC spatial data support the enrichment of SFRP2-associated fibroblast programs within the tumor-associated stromal microenvironment, while not implying direct single-cell contact between individual fibroblasts and tumor cells.

To explore potential signaling interactions involving this fibroblast state, we performed ligand–receptor analysis using BCC scRNA-seq data. This analysis identified fibroblast-associated WNT-related communication, including an SFRP2-LRP5/6 axis (Figure 3B). Because ligand–receptor inference is correlative, we interpret this result as evidence for a candidate fibroblast-associated WNT signaling axis rather than proof of pathway activation.

We next mapped SFRP2 expression, inferred SFRP2-positive fibroblast abundance, BCC-associated signal, and WNT5A expression across healthy control and BCC tissue sections (Figure 3C). SFRP2-associated fibroblast signals were enriched in the stromal regions surrounding the tumor, with adjacent or partially overlapping WNT5A expression in tumor-associated areas. These spatial patterns support the presence of SFRP2-associated fibroblast programs near WNT-active tumor niches in BCC.

Since Visium is limited by spot-level resolution, we further analyzed BCC *in situ* sequencing data from the spatial skin atlas. Since SFRP2 was excluded from the ISS panel, we therefore used scRNA-seq data to identify ISS panel genes that were positively correlated with SFRP2 expression in BCC fibroblasts and combined representative genes into a mean z-scored SFRP2-associated fibroblast proxy score (Supplementary Figure S4B). Projection of this proxy score onto BCC ISS sections showed SFRP2-proxy-high fibroblast signals near basal/tumor compartment cells, providing orthogonal higher-resolution spatial support for the Visium-based observation (Supplementary Figure S4C).

### SFRP2-associated fibroblast programs are enriched at SCC tumor–stroma interfaces

**Figure 4:**
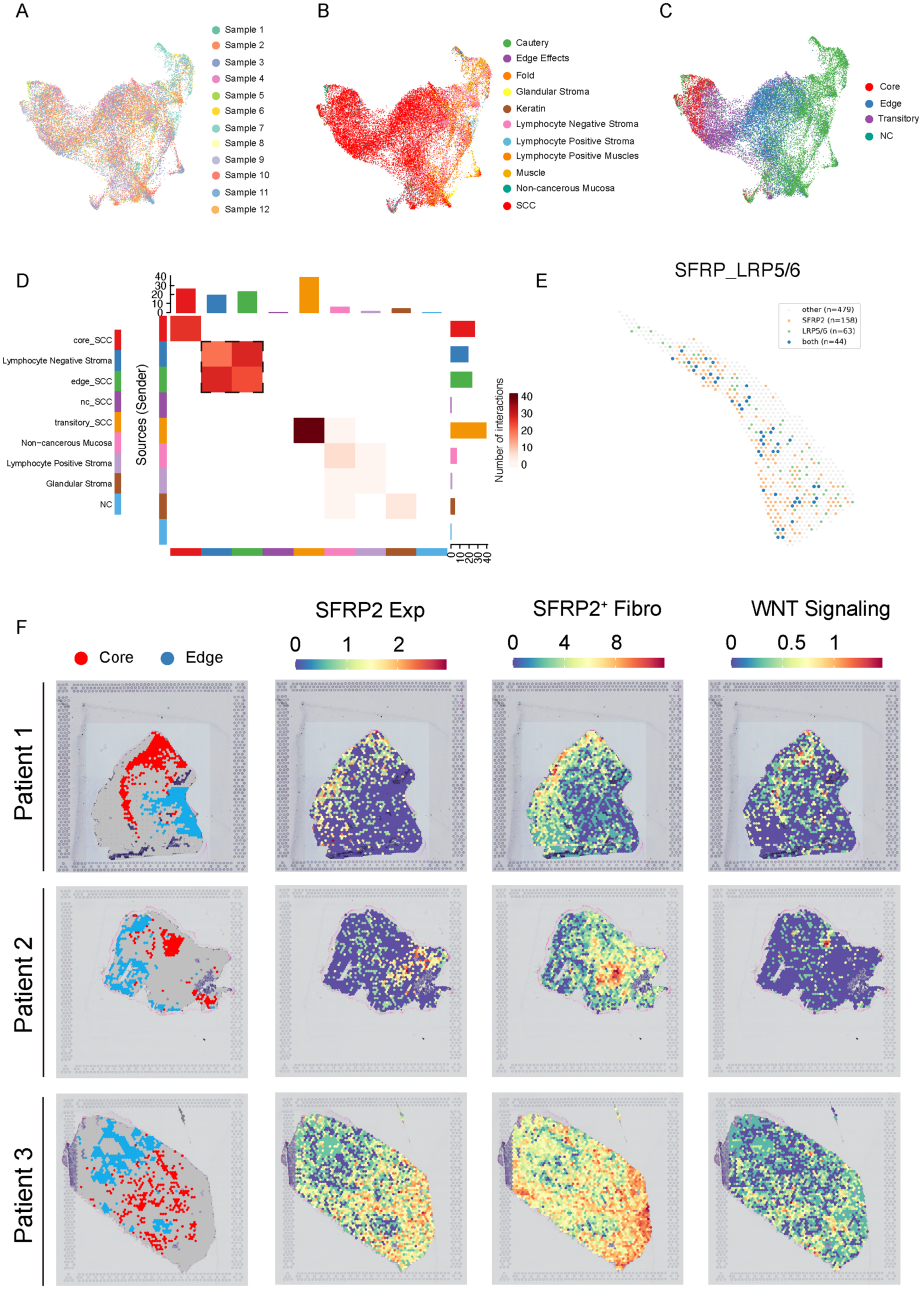
SFRP2-positive fibroblast programs and WNT signaling are enriched at SCC tumor–stroma interfaces. **(A–C)** UMAP/spatial embeddings of SCC Visium spots colored by sample identity, pathological annotation, and aggregated tumor zone, respectively. **(D)** Spatial proximity heatmap showing neighborhood relationships among SCC tumor zones and stromal compartments. **(E)** Spatial visualization of the SFRP2-LRP5/6 ligand–receptor axis in SCC. **(F)** Representative Visium spatial maps from three SCC patients showing tumor core/edge annotation, SFRP2 expression, inferred SFRP2-positive fibroblast abundance, and WNT signaling score.

To determine whether SFRP2-associated fibroblast programs were also present in squamous cell carcinoma (SCC), we analyzed SCC Visium spatial transcriptomic data. UMAP/spatial embeddings separated spots by sample identity, pathological annotation, and aggregated tumor zone, indicating substantial spatial heterogeneity across SCC samples (Figures 4A–C). Spatial proximity analysis showed organized neighborhood relationships between SCC tumor zones and adjacent stromal compartments (Figure 4D), consistent with a structured tumor–stroma interface.

We next examined fibroblast-associated WNT-related signaling in SCC. Ligand–receptor analysis identified an SFRP2-LRP5/6 signaling axis involving fibroblast-associated SFRP2 and receptor expression in neighboring tumor-associated compartments (Figure 4E). Since ligand–receptor analysis is inferential, we interpret this result as a candidate fibroblast-associated WNT signaling interaction rather than direct evidence of pathway activation.

We then mapped SFRP2 expression, inferred SFRP2-positive fibroblast abundance, and WNT signaling scores across representative SCC tissue sections (Figure 4F). SFRP2-associated fibroblast signals were enriched in tumor core or tumor edge-adjacent stromal regions, where WNT signaling scores were also elevated. These spatial patterns suggest that SFRP2-associated fibroblast programs are positioned near WNT-active tumor–stroma niches in SCC.

**Figure 5:**
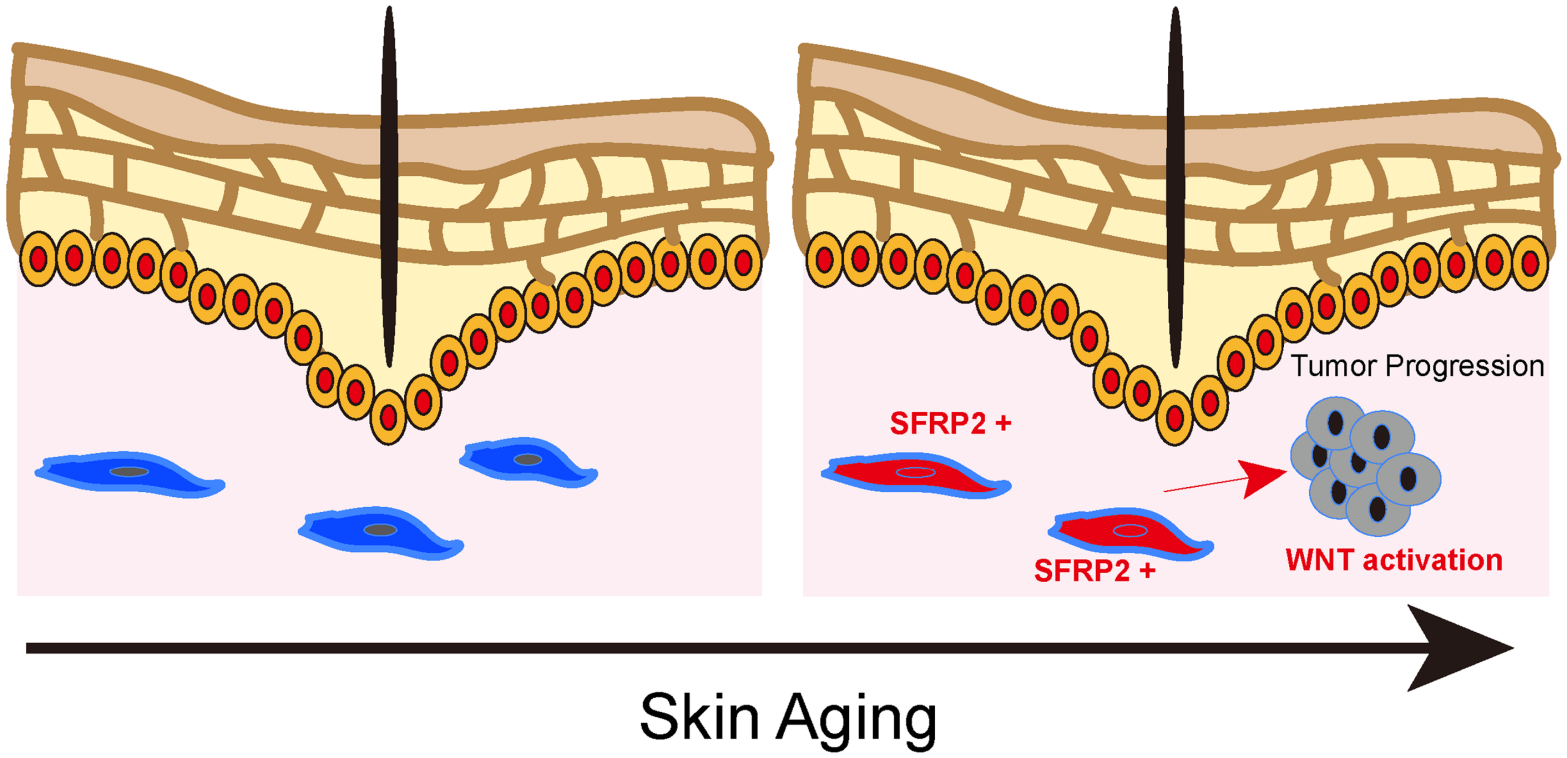
Working model of aging-associated fibroblast remodeling and increased tumor vulnerability. Schematic model illustrating the proposed accumulation of SFRP2-expressing fibroblasts during skin aging and their enrichment in tumor-associated stroma. The SFRP2-associated fibroblast program is spatially associated with WNT-active tumor or tumor-adjacent compartments, contributing to a tumor-permissive microenvironment.

To further evaluate transcriptional programs associated with SFRP2-expressing fibroblasts, we stratified SCC fibroblasts into SFRP2-high and SFRP2-low subsets. SFRP2-high fibroblasts showed elevated expression of fibroblast and matrix-associated genes, including COL1A1, COL1A2, FN1, POSTN, FAP, and DCN, as well as matrix-remodeling genes MMP2 and MMP14 and growth factor−/angiogenesis-related genes FGF7 and VEGFB (Supplementary Figure S5B). Spatial Visium maps showed that these SFRP2-associated fibroblast markers and mechanism-related genes were enriched in tumor-associated stromal regions (Supplementary Figure S5C). Together, these findings support a model in which age-associated accumulation of SFRP2-expressing fibroblasts may contribute to a tumor-permissive stromal microenvironment during skin cancer development (Figure 5).

## Discussion

Aging is increasingly recognized as a key contributor to cancer susceptibility, not only through the accumulation of genetic mutations but also via profound changes in the tissue microenvironment (28, 29). In this study, we integrated single-cell and spatial transcriptomics to dissect age-associated alterations in human skin and skin cancers. Our results highlight marked shifts in cellular composition and gene expression programs with age. Importantly, the decision to focus on fibroblasts was guided by the age-adjusted survival analysis: among the evaluated cell-type-specific aging programs, the fibroblast aging signature showed the clearest association, in which increased signature activity was associated with poorer survival independent of chronological age. These findings implicate fibroblasts as central players in creating a tumor-permissive aged microenvironment (Figure 5).

Among fibroblast populations, we identified an SFRP2-positive fibroblast state enriched in aged skin and further expanded in basal cell carcinoma (BCC). This finding is best interpreted in light of Tabib et al., who identified SFRP2/DPP4-positive fibroblasts as one of the two major fibroblast populations in normal human skin (28). In that physiological context, these cells were described as small, elongated fibroblasts located between collagen bundles and enriched for extracellular matrix-associated genes, including type I collagen, fibrillin, fibronectin, and DPP4, supporting a role in matrix deposition and dermal matrix organization (28). Thus, SFRP2 expression itself does not define an aging- or tumor-specific fibroblast population; rather, it marks a pre-existing homeostatic matrix-organizing fibroblast state.

These normal functions provide a conceptual bridge between homeostasis and disease. Fibroblasts that maintain dermal matrix architecture under physiological conditions may become increasingly prominent in aged skin, where chronic stromal remodeling, altered matrix turnover, and reduced tissue resilience can shift a homeostatic extracellular matrix program toward a remodeling-prone state. In this model, aging does not create a completely new fibroblast identity but rather quantitatively expands and potentially redirects an existing SFRP2-positive ECM-organizing program. Such a shift may help explain why aged skin is more permissive to tumor development: excessive or spatially reorganized matrix-producing fibroblasts can alter tissue architecture and provide paracrine cues that support tumor–stroma interactions.

The disease relevance of this homeostatic-to-pathological transition becomes apparent in tumor tissues. In BCC, SFRP2-positive fibroblasts were further enriched and spatially associated with tumor regions. Although SFRP2 has traditionally been viewed as a WNT antagonist, it has also been shown to activate non-canonical WNT/Ca2 + signaling in cancer contexts and to promote tumor angiogenesis via Fzd5/NFAT pathways. Consistent with this context-dependent activity, our spatial and ligand–receptor analyses suggest that SFRP2-positive fibroblasts may contribute to a tumor-supportive microenvironment by coupling ECM remodeling with WNT pathway modulation and angiogenic signaling. This interpretation links the physiological ECM-organizing role of SFRP2-positive fibroblasts described by Tabib et al. to our observation that the same fibroblast program becomes enriched in aging and tumor contexts.

Importantly, aging signatures varied considerably across cell types and tumor types. While fibroblast aging was prominent in BCC, immune and endothelial compartments displayed distinct aging trajectories. For example, endothelial cells in aged skin exhibited elevated angiogenic programs, including VEGF signaling, known to enhance tumor vascularization. In contrast, immune aging was more pronounced in melanoma and squamous cell carcinoma (SCC), consistent with the higher immunocyte infiltration and immunosuppressive microenvironments observed in those cancers. These differences reinforce the need for tumor type-specific strategies when targeting aging-associated pathways.

Spatial transcriptomics was essential for resolving cell localization and interaction networks, enabling us to map the enrichment and spatial clustering of SFRP2-positive fibroblasts near tumor sites. This integrative approach helped overcome the limitations of single-cell data in estimating abundance and provided a contextual understanding of how aging fibroblasts interact with surrounding cells to influence cancer behavior.

Therapeutically, targeting SFRP2-positive fibroblasts or modulating aberrant WNT signaling may offer novel strategies for age-associated skin cancers (29). SFRP2 itself may serve as a biomarker to stratify patients who might benefit from such interventions. More broadly, this study underscores the importance of incorporating aging biology into cancer research frameworks. Future studies should investigate whether similar fibroblast-driven mechanisms operate in other aging tissues and cancer types and explore interventions that mitigate age-induced stromal changes to improve cancer outcomes.

### Limitations of the study

While our study offers a comprehensive computational analysis of age-associated fibroblast dynamics and their potential link to skin cancer progression, we acknowledge that its primary limitation lies in the absence of direct experimental validation. Specifically, although our integrative analyses revealed a robust and conserved enrichment of SFRP2^+^ fibroblasts in aged skin and tumor contexts, the biological function and causal relevance of this population remain to be experimentally validated.

## Data Availability

The original contributions presented in the study are included in the article/Supplementary material, further inquiries can be directed to the corresponding author.

## Supplementary material

SUPPLEMENTARY TABLE S1 Cell-type signature for scRNA-seq annotation.

SUPPLEMENTARY TABLE S2 Cell-type-specific aging signatures.

SUPPLEMENTARY TABLE S3 Spatial statistics.

SUPPLEMENTARY TABLE S4 Figure 1B trajectory heatmap gene list.

SUPPLEMENTARY TABLE S5 Figure 1D secreted signaling ligand-receptor reference set.

SUPPLEMENTARY TABLE S6 Figure 3B ligand-receptor gene list.

SUPPLEMENTARY FIGURE S1 Integration benchmarking and age-associated cell-composition changes in healthy-skin scRNA-seq data.

SUPPLEMENTARY FIGURE S2 Cell-type marker validation and age-associated differential expression across healthy-skin cell types.

SUPPLEMENTARY FIGURE S3 Age-associated TNF, CXCL, and MIF signaling and immune-cell aging gene programs.

SUPPLEMENTARY FIGURE S4 Validation of aging signatures and ISS proxy analysis of SFRP2-associated fibroblasts.

SUPPLEMENTARY FIGURE S5 SFRP2 expression and mechanism-associated gene programs in fibroblasts.
