# Supplementary figures and images for: Integrated single-cell and spatial transcriptomic analyses reveal an aging-associated fibroblast subtype linked to tumor progression in human skin
Source: Front Med (Lausanne). 2026 Jul 8;13:1800947. doi: 10.3389/fmed.2026.1800947 (PMC13393490; doi:10.3389/fmed.2026.1800947)

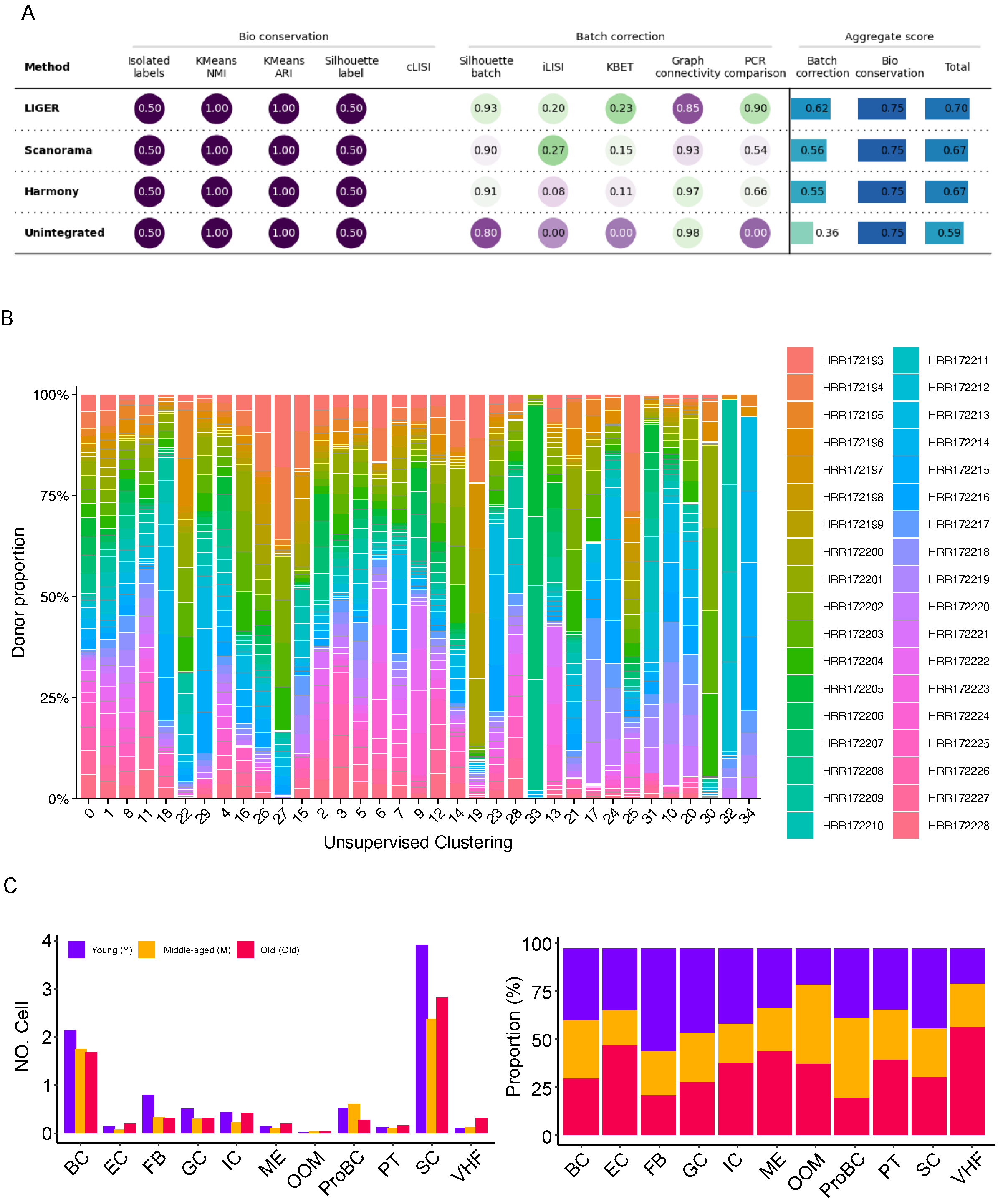

Supplement: SUPPLEMENTARY FIGURE S1 — Integration benchmarking and age-associated cell-composition changes in healthy-skin scRNA-seq data. [file Image_1.tif]

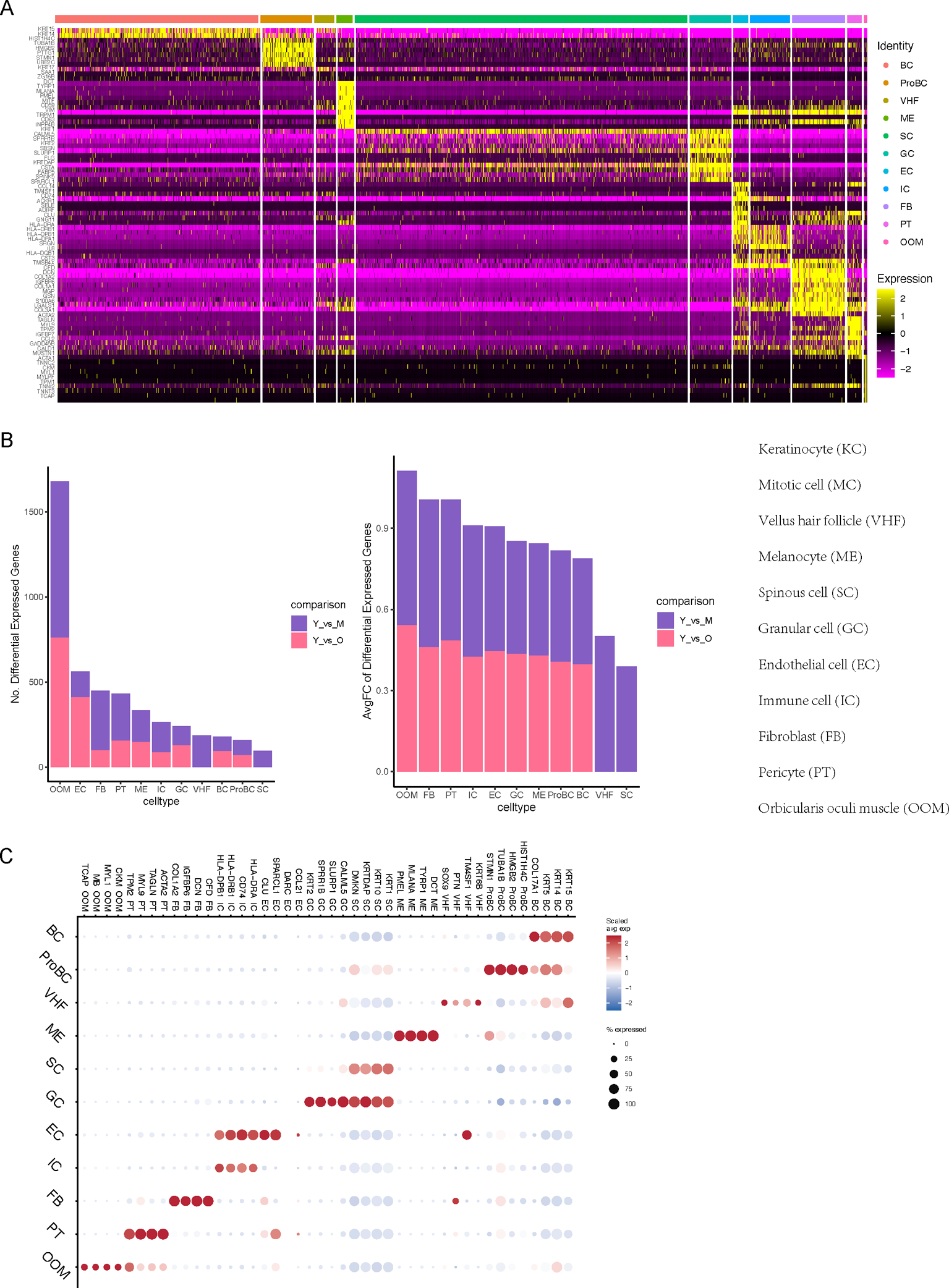

Supplement: SUPPLEMENTARY FIGURE S2 — Cell-type marker validation and age-associated differential expression across healthy-skin cell types. [file Image_2.tif]

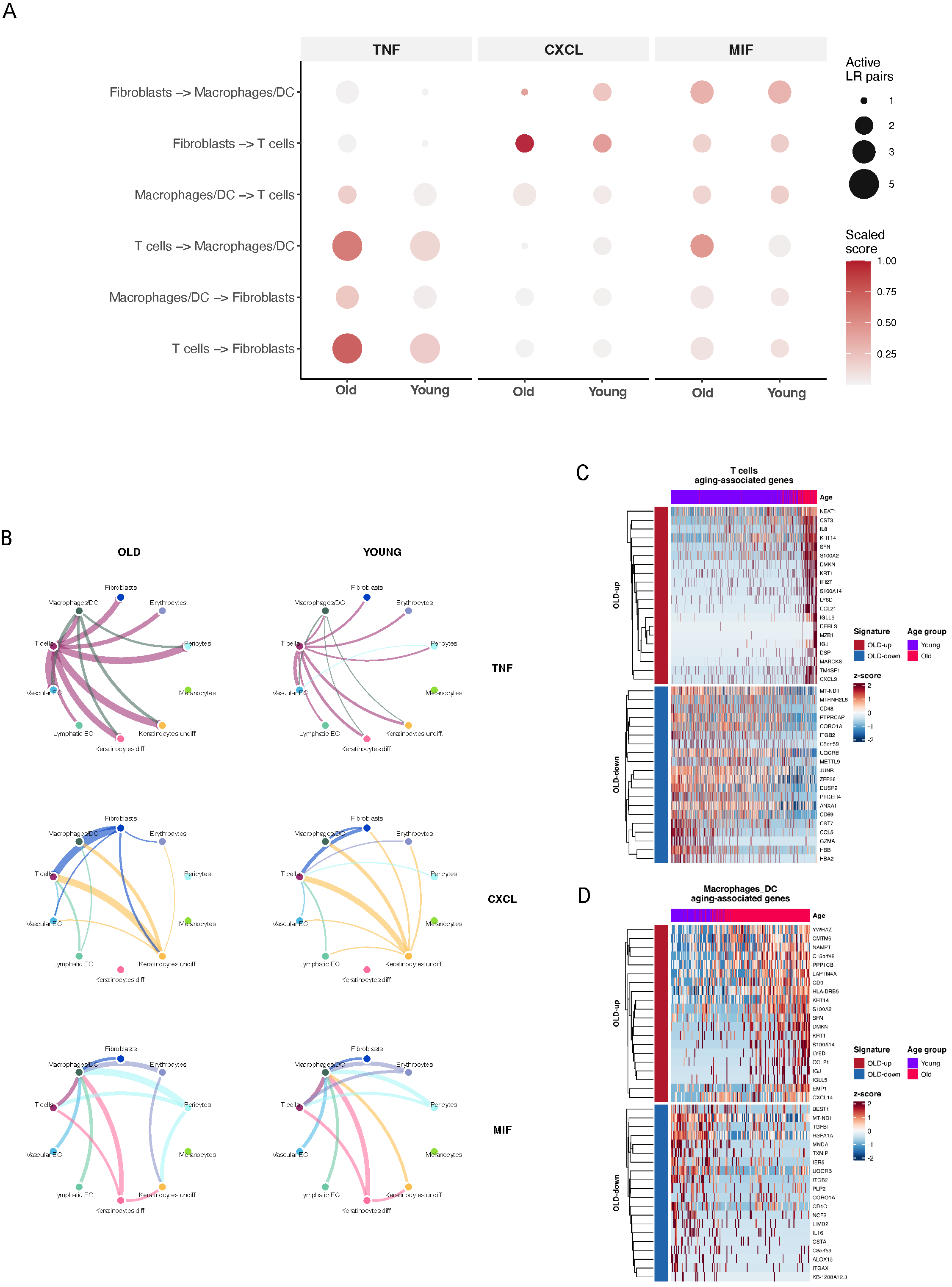

Supplement: SUPPLEMENTARY FIGURE S3 — Age-associated TNF, CXCL, and MIF signaling and immune-cell aging gene programs. [file Image_3.tif]

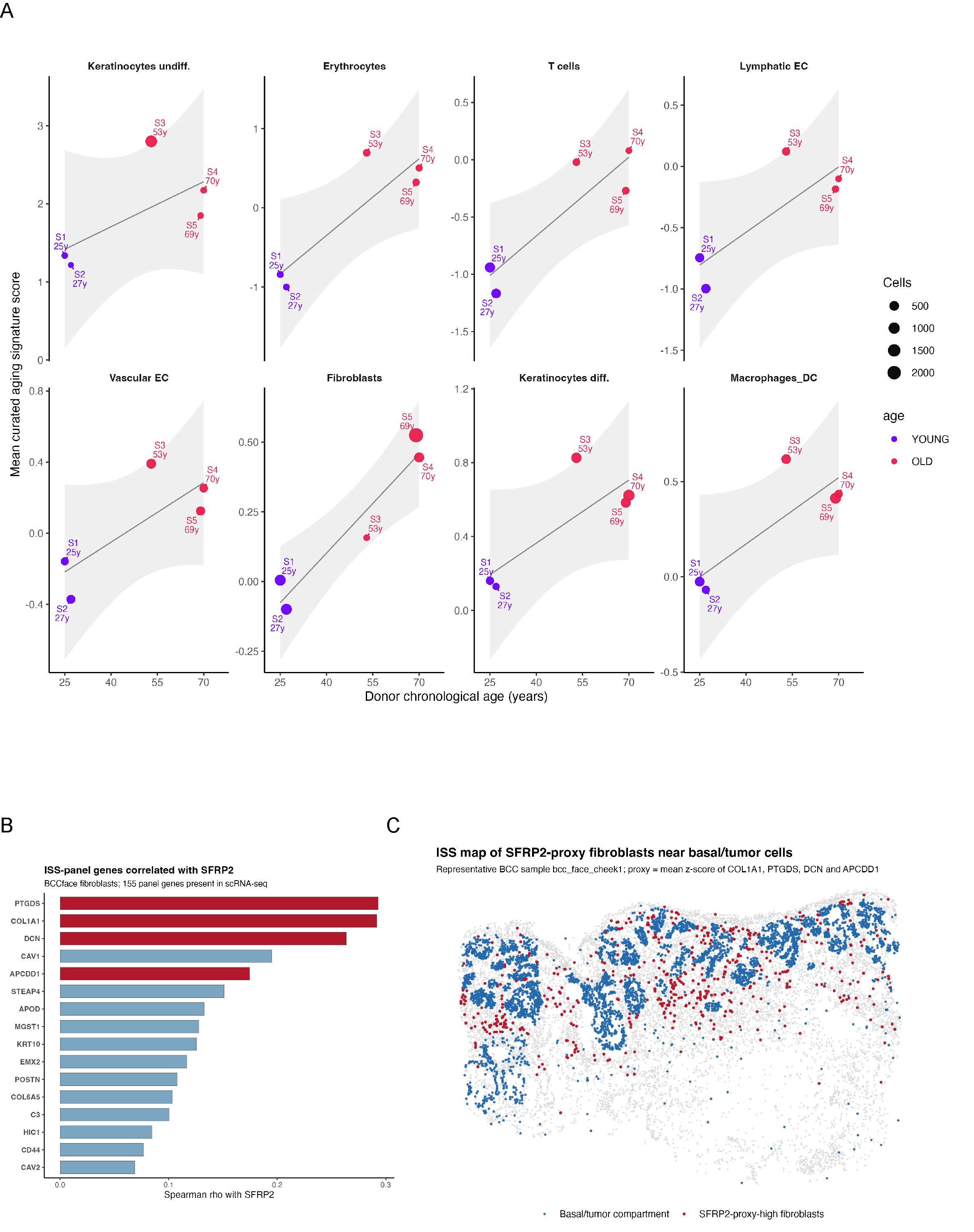

Supplement: SUPPLEMENTARY FIGURE S4 — Validation of aging signatures and ISS proxy analysis of SFRP2-associated fibroblasts. [file Image_4.tif]

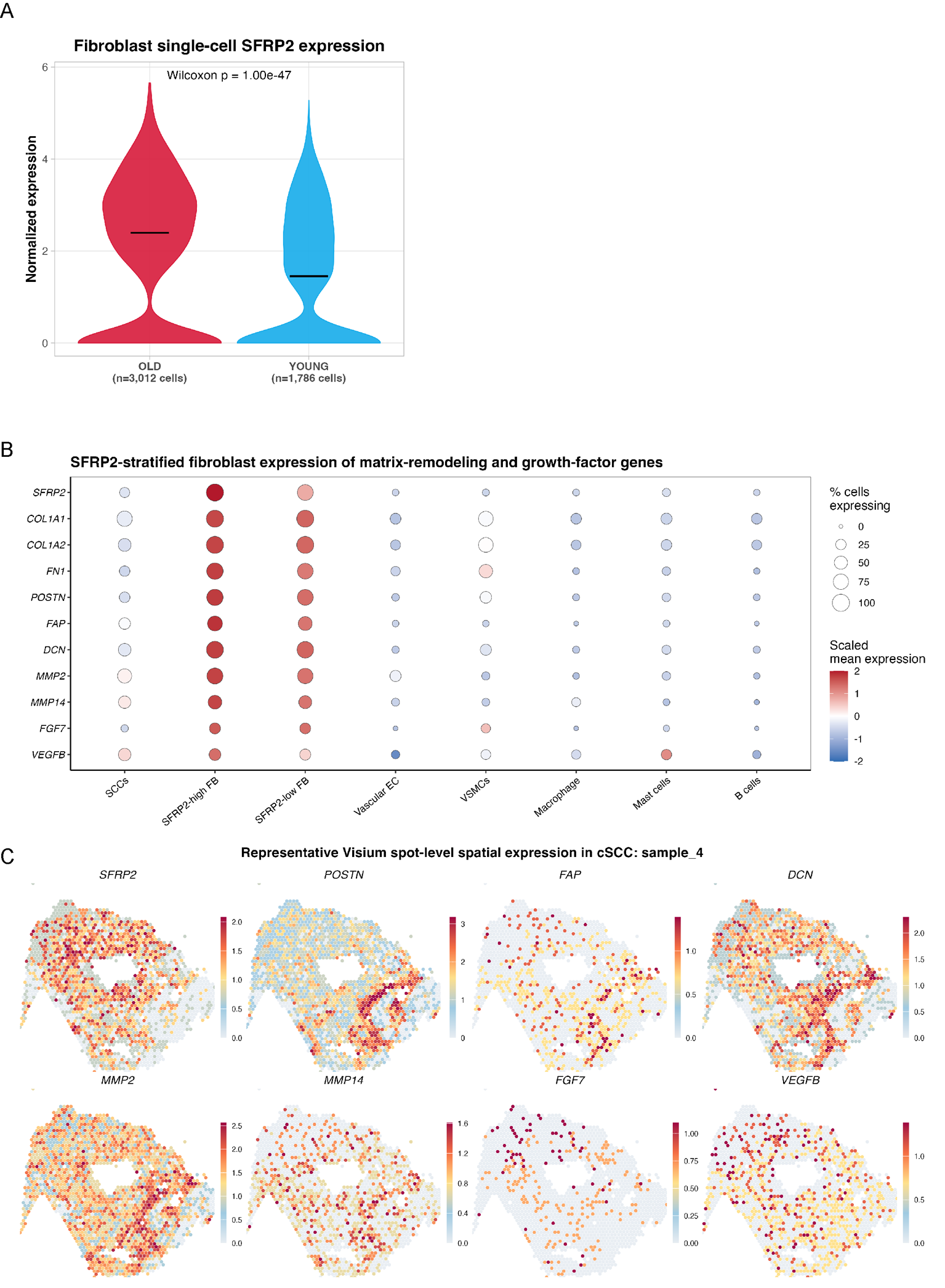

Supplement: SUPPLEMENTARY FIGURE S5 — SFRP2 expression and mechanism-associated gene programs in fibroblasts. [file Image_5.tif]
